# Insights into complementary exposomic targeted analysis and suspect screening approaches: a case study examining human serum for chemicals with LC-IMS-MS

**DOI:** 10.1039/d6va00088f

**Published:** 2026-04-17

**Authors:** James N. Dodds, Nikki Barlow, Kara M. Joseph, Sarah J. Rehm, Weihsueh A. Chiu, Gang Han, Yu-Syuan Luo, Kangmin Zhu, Warren Casey, Ivan Rusyn, Erin S. Baker

**Affiliations:** a Department of Chemistry, University of North Carolina at Chapel Hill Chapel Hill North Carolina 27514 USA erinmsb@unc.edu; b Department of Veterinary Physiology and Pharmacology, Texas A&M University College Station TX 77843 USA; c Department of Epidemiology & Biostatistics, Texas A&M University College Station TX 77843 USA; d Institute of Food Safety and Health, College of Public Health, National Taiwan University Taipei 100025 Taiwan; e John P. Murtha Cancer Center Research Program, Uniformed Services University of the Health Sciences Bethesda MD 20817 USA; f Division of Epidemiology and Biostatistics, Department of Preventive Medicine and Biostatistics, Uniformed Services University of the Health Sciences Bethesda MD 20814 USA; g Henry Jackson Foundation for the Advancement of Military Medicine Bethesda MD 20817 USA; h Division of Program Coordination, Planning, and Strategic Initiatives, U. S. National Institutes of Health Bethesda MD 20892 USA

## Abstract

Although PFAS exposure is widespread in the general population, concern is heightened for individuals with unique occupational exposure scenarios. Accordingly, the PROject for Military Exposures and Toxin History Evaluation in US service members (PROMETHEUS) study is evaluating whether serum chemical exposure profiles correlate with cancer incidence in large cohorts (typically hundreds of samples per analysis) among military service members who may experience distinct occupational and environmental exposures. Here we describe analytical workflow development and results from a pilot subset (*n* = 36) of human serum samples using an integrated targeted and suspect-screening LC-IMS-MS platform. Serum (50 µL) was extracted by acid-assisted protein precipitation with isotopically labeled internal standards, concentrated, and analyzed by LC coupled to an Agilent 6560 IM-QTOF. Targeted PFAS quantitation was performed using matrix-matched calibration curves and was benchmarked against NIST SRM 1957 to assess method accuracy. Across the samples, the targeted panel captured predominantly legacy PFAS as anticipated noting their prevalence in prior studies (*e.g.*, PFOS, PFOA, 8 : 2 FTS, *N*-MeFOSAA, *etc.*). Ultra-short-chain PFAS presented class-specific analytical challenges; trifluoromethanesulfonic acid (TFMS) was observed, whereas trifluoroacetic acid (TFA) eluted near the void volume and exhibited pronounced clustering in the ion mobility dimension, precluding reliable quantitation. In parallel, CCS-based mobility filtering supported suspect screening against an exposomic library (∼1100 entries) to expand detectable chemical space beyond targeted PFAS. Suspect screening yielded 49 non-PFAS candidates meeting accurate mass and CCS agreement criteria, and correlation analysis recapitulated expected co-exposure groupings among legacy PFCAs/PFSAs and structurally related suspect analytes. Collectively, these results establish a scalable, CCS-informed LC-IM-MS workflow for integrated targeted PFAS quantitation and exposomic suspect screening, enabling higher-powered association testing in the full set of PROMETHEUS samples and other large-scale human biomonitoring studies.

## Abstract 2

Here we report a scalable analytical workflow that integrates targeted PFAS quantitation with collision cross section (CCS)enabled LC-IMS-MS suspect screening to improve selectivity and annotation confidence incomplexserum matrices.

## Graphical abstract

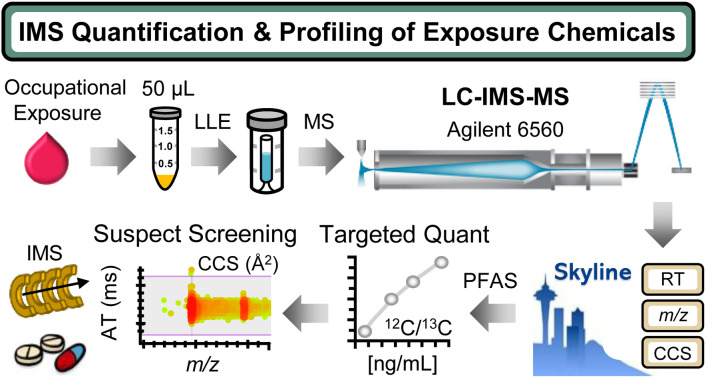

**Environmental significance**

Per- and polyfluoroalkyl substances (PFAS) and other xenobiotic contaminants pose a persistent environmental health challenge because human exposure is widespread, chemical space is rapidly expanding, and many emerging compounds lack analytical standards needed for confident identification. In this work, we demonstrate an integrated LC-ion mobility spectrometry-mass spectrometry (LC-IMS-MS) workflow that couples targeted PFAS quantitation with collision cross section (CCS) enabled suspect screening to improve selectivity and annotation confidence in complex serum matrices. Using low-volume archived serum (50 µL) from the Department of Defense Serum Repository (DoDSR), we benchmark quantitative performance against NIST SRM 1957 and show how CCS-based mobility filtering can reduce isomeric ambiguity and expand chemical coverage beyond traditional targeted panels. By tentatively annotating 49 non-PFAS xenobiotics alongside robust legacy PFAS quantitation in a pilot cohort (*n* = 36), this study highlights how IMS-derived CCS descriptors are transferable across laboratories and can support scalable, standardized biomonitoring and exposomic assessments that connect environmental contaminants to human health outcomes.

## Introduction

Per- and polyfluoroalkyl substances (PFAS) are persistent and ubiquitous environmental contaminants associated with widespread human exposure and increasing concern for long-term adverse health outcomes.^1–3^ PFAS are widely used as durable surfactants in household and industrial applications, where their high degree of fluorination and strong carbon-fluorine (C–F) bonds impart chemical resistance and thermal stability, contributing to environmental persistence and bioaccumulation.^4^ Epidemiologic studies, including those by the C8 Science Panel, have associated PFAS exposure with adverse outcomes including elevated serum cholesterol,^5,6^ thyroid disease,^7^ and increased incidence risk of multiple cancers.^8,9^ Moreover, biomonitoring surveys suggest that the majority of U.S. residents have measurable PFAS in blood, reflecting widespread exposure through inhalation, drinking water, diet, and consumer products.^10,11^

In response to the concerns about both exposure and potential adverse effects, several regulatory agencies have encouraged manufacturers to phase out production of traditionally used long-chain perfluoroalkyl sulfonic acids (PFSAs) and perfluoroalkyl carboxylic acids (PFCAs), including perfluorooctane sulfonic acid (PFOS) and perfluorooctanoic acid (PFOA).^12^ However, continued commercial demand has driven the development and use of “emerging PFAS” or “replacement chemistries,” including ether-linked analogs (*e.g.*, hexafluoropropylene oxide dimer acid, HFPO-DA/GenX) and chlorinated derivatives.^13,14^ This rapidly expanding chemical space necessitates development of the analytical methods beyond conventional targeted liquid chromatography-mass spectrometry (LC-MS), which typically quantifies a limited set of analytes.^15^ The U.S. Environmental Protection Agency (EPA) has estimated the existence of ∼14 000 PFAS,^16^ while broader definitions (*e.g.*, the Organisation for Economic Co-operation and Development, OECD) encompass even greater number of fluorinated structures.^17^ Accordingly, targeted analytical chemistry workflows are now increasingly complemented by suspect screening analysis (SSA) and non-targeted analysis (NTA) workflows, wherein full-scan data are retrospectively interrogated for presence of candidate PFAS and other chemical classes for confirmatory identification.^18,19^

As the adoption of suspect screening and non-targeted workflows continue to increase, additional methods are needed to improve feature annotation and reduce false positives. Ion mobility spectrometry (IMS) provides an orthogonal, gas-phase separation based on each ion's size, shape, and charge and reports this property as the analyte's collision cross section (CCS, Å^2^), a quantitative measure of ion-neutral effective surface area.^20^ IMS adds enhanced selectivity in complex matrices and improves discrimination of isomeric species while operating on the millisecond timescale, enabling seamless integration into conventional LC-MS workflows.^21–23^ For temporally-based IMS separations, each ion's drift time is highly dependent on laboratory conditions (*e.g.*, temperature, pressure and applied voltage), and hence these observed values are converted to standardized CCS values, which serve as transferable molecular descriptors that can be shared across instruments and laboratories.^24–26^ This process enables the collation and dissemination of CCS libraries across IMS research efforts and increases confidence in both targeted and suspect-screening workflows.^27^

While it is widely known that general population exposure to PFAS may occur *via* ingestion and inhalation of contaminated media, certain occupational categories may experience additional PFAS exposure burden, including firefighters and military personnel who may work with aqueous film-forming foams (AFFF).^28^ To better understand potential links between military personnel exposures and health outcomes, the PROject for Military Exposures and Toxin History Evaluation in US service members (PROMETHEUS) initiative was established to collect and study specimens from the Department of Defense Serum Repository (DoDSR), an extension of the Armed Forces Health Surveillance Center (AFHSC), which contains >70 million serum samples collected longitudinally from >10 million service members since 1986. To enable reproducible analyses of these and other samples from large-scale human biomonitoring studies, here we report analytical workflow development and preliminary findings from a pilot subset of samples (*n* = 36), utilizing LC-IMS-MS to integrate targeted quantitative PFAS profiling with complementary suspect screening exposomic analysis. This combined strategy improves selectivity in complex serum matrices, expands detectable chemical space beyond targeted panels, and establishes a scalable platform for analysis of the full PROMETHEUS cohort.

## Experimental methods

### Human study samples

For this study, archived serum specimens were selected from the broader DoDSR repository with approval from the Uniformed Services University of the Health Sciences and the Walter Reed National Military Medical Center Institutional Review Boards under IRB DBS.2024.761 following appropriate laws and institutional guidelines.^29^ Subjects were initially notified by the AFHSC using an informative letter to describe the study purpose, procedures, a questionnaire packet and appropriate informed consent documentation for study consideration. Patients responding in the affirmative were carried forward with sample collection. Samples were curated and stored frozen at a centralized repository and shipped overnight on dry ice to participating laboratories for analysis. Because aliquots available for analyses were limited (typically ≤100 µL per sample), maximizing extraction efficiency and analytical performance from low-volume serum was a primary driver of method development.

### Serum extraction protocol

**Fig. 1:**
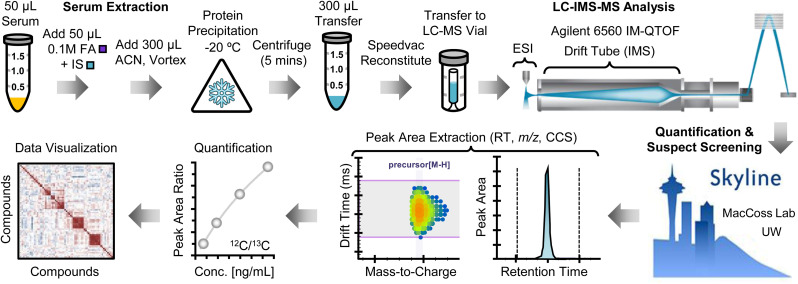
Overview of the serum extraction and LC-IMS-MS workflow used for targeted PFAS quantitation and CCS-enabled suspect screening in the PROMETHEUS pilot study dataset. Formic acid, acetonitrile, and internal standard are denoted by FA, ACN and IS, respectively.

After evaluating reported serum PFAS workflows, a modified extraction adapted with internal guidance from the U.S. Environmental Protection Agency (EPA) was implemented.^30^ An overview is provided in Fig. 1 (detailed procedures are featured in the SI, Fig. S1). Briefly, 50 µL of serum was thawed at room temperature and acidified with 50 µL of 0.1 M formic acid containing isotopically labeled internal standards (MPFAC–HIF–ES, Wellington Laboratories, Guelph, ON). Proteins were precipitated by addition of 300 µL acetonitrile followed by incubation at −20 °C, then samples were centrifuged and 300 µL supernatant was transferred to a 2 mL microcentrifuge tube. Extracts were concentrated by speed-vacuum and reconstituted in 75 µL methanol buffered with ammonium acetate prior to LC-IMS-MS analysis.

Method accuracy was assessed using NIST SRM 1957 human serum, which provides verified concentrations for select legacy PFAS, and overall process performance was evaluated in line with prior PFAS studies *via* process efficiency assessments. Matrix-matched calibration curves were generated by spiking the PFAC30PAR mixture (Wellington Laboratories; 30 PFAS spanning legacy and replacement chemistries) into fetal bovine serum (FBS) across 0.001–100 ng mL^−1^, followed by extraction using the same protocol applied to biological samples and SRM 1957. To assess performance for ultra-short/short-chain targets, five short-chain analytes (≤C3) representing PFCA/PFSA classes (TFA, PFPRA, TFMS, PFEtS, PFPrS) were independently spiked into FBS and processed identically to evaluate retention, sensitivity, and potential detectability in study serum samples.

### LC-IMS-MS data acqusisiton

Extracted samples (4 µL) were injected onto an Agilent 1290 LC for chromatographic separation using a recently developed intrinsically base-deactivated (IBD) C18 column (Restek, Bellefonte, PA).^31^ This stationary phase incorporates a polar functional group within the C18 ligand to improve retention and selectivity for more polar PFAS while maintaining traditional reversed-phase behavior. Separations were performed using a 12 min gradient (including re-equilibration) with mobile phase A consisting of water buffered with ammonium formate and 0.1% formic acid, and mobile phase B consisting of acetonitrile. This method provided adequate resolution of linear and branched PFAS within a short run time; an example extracted ion chromatogram (EIC) for PFAS standards is shown in Fig. S2 alongside extended chromatographic settings (Fig. S3).

Collision cross section (CCS) calibration was performed using the Agilent Tune Mix and the single-field approach as described in detail by Stow *et al.*^32^ In brief, tune mix ions were infused under identical IMS-MS conditions as study samples to generate a calibration function relating arrival time to known CCS.^20,32^ This relationship was then used to report CCS values for detected features and to subsequently translate library CCS values from existing databases into expected arrival times for CCS-based mobility filtering. This approach was utilized for both targeted PFAS quantitation and suspect-screening candidate evaluation, and full LC-IMS-MS acquisition parameters are provided in Fig. S3.

### Data analysis and visualization

Agilent .d files were demultiplexed using the PNNL PreProcessor (v4.0) to deconvolute multiplexed ion mobility data and were CCS-calibrated using the single-field workflow.^33^ Processed files were imported into Skyline^34–36^ initially for targeted quantitative PFAS analysis and subsequently for CCS-informed suspect screening analysis (SSA) using both previously published PFAS studies^37–39^ and the exposomic suspect library (∼1100 negative-mode entries) reported by Teri *et al.*^40^ Candidate signals were prioritized based on agreement in accurate mass, CCS/arrival time, and isotopic envelope, and as such are denoted with Level 3 confidence following the guidance published by Boatman *et al.*^41^ PFAS concentrations, peak areas, and suspect-screening feature areas were exported from Skyline to Microsoft Excel for data analysis and visualization.

#### Targeted MS

**Fig. 2:**
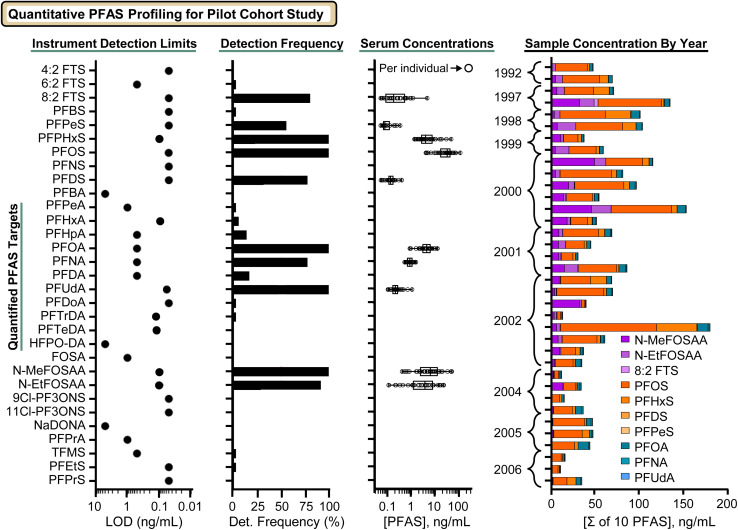
Quantitative PFAS profiling in a pilot set of samples of serum collected from military service personnel (*n* = 36). From left to right, panels summarize: (1) instrument limits of detection (LODs) determined from matrix-matched calibration curves; (2) detection frequency across serum samples with corresponding concentration distributions for each quantified PFAS; (3) detected PFAS concentrations across the dataset, where each circle denotes an individual serum sample and (3) total (summed) PFAS concentrations per individual sample, annotated by year of collection and individual PFAS (inset).

Targeted quantitation was performed by comparing serum analytes to matrix-matched standards extracted in triplicate, using retention time, CCS, and accurate mass as orthogonal data filters. Limits of detection (LOD) were defined as the lowest concentration demonstrating a monotonic increase in signal with concentration and exceeding 3σ of the method blank. Limits of quantification (LOQ) were defined as 10*σ* above the blank, <20% RSD across replicate extractions, and ≥ LOD.^42^ LODs ranged from 0.05–100 ng mL^−1^ depending on analyte, with lower LODs generally observed for legacy long-chain PFCAs/PFSAs and fluorotelomer sulfonates (FTSs), and higher LODs for short-chain analytes and select ether-linked replacement chemistries (Fig. 2). Peak areas were normalized to isotopically labeled internal standards, and calibration curves were fit using quadratic regression with 1/*x* or 1/*x*^2^ weighting as appropriate. When detector saturation was observed at 100 ng mL^−1^ for highly responsive analytes (*e.g.*, 4 : 2 FTS, TFMS), the saturated point was excluded from curve fitting. Representative calibration curves and LOD/LOQ/ULOQ criteria are provided in Fig. S4, and analyte-specific values are reported in the SI spreadsheets.

#### Suspect screening analysis (SSA)

Suspect screening efforts focused on non-PFAS chemicals compiled by Teri *et al.*,^40^ which reports CCS values for 2144 unique chemicals prioritized from the EPA ToxCast program. Because data were acquired in ESI-, the relevant library subset contained ∼1100 entries, which were screened using agreement in accurate mass and CCS-based mobility filtering. Mass measurement accuracy for tentatively identified matches was typically ≤5 ppm relative to the proposed molecular formula, and candidate isomers were evaluated by their expected arrival-time fit to the library CCS value. Suspect feature annotations are therefore tentatively identified based on accurate mass, CCS agreement and isotopic envelope, as the database does not include retention time or MS/MS fragmentation metadata precluding higher level annotations beyond Level 3 confidence.^41^ Raw data for both targeted PFAS quantification and SSA is provided for public view *via* the Panorama Dashboard at https://panoramaweb.org/PrometheusPilot.url.

#### Quality control and method accuracy

Quantitative accuracy was evaluated using NIST SRM 1957 human serum, comparing measured concentrations to assigned values for PFOA, PFDA, PFUdA, PFHxS, and PFOS. Observed accuracies ranged from 101–128%, within a typical ±30% acceptance threshold (Fig. S5). Extraction process efficiencies were calculated for the PFAC30PAR targets and generally ranged between 70-130% as summarized in Fig. S6.

## Results and discussion

### Quantitative PFAS assessment

Targeted quantitation was performed for 35 PFAS, spanning legacy perfluoroalkyl carboxylic acids (PFCAs), perfluoroalkyl sulfonic acids (PFSAs), fluorotelomer sulfonates (FTSs), sulfonamide derivatives, ether-linked replacement chemistries (*e.g.*, HFPO-DA/GenX, NaDONA), and select ultra-short-chain PFAS. Of the targeted analytes, trifluoroacetic acid (TFA) could not be reliably characterized using the LC-IMS-MS method developed herein. Although the intrinsically base-deactivated (IBD) C18 method provided strong selectivity for many PFAS classes, TFA was poorly retained and eluted around the chromatographic void volume, potentially reflecting differences between serum matrix extracts and the aqueous matrices evaluated in the manufacturer application note. In addition, while TFA ionizes efficiently in negative-mode ESI, the corresponding mobility signal exhibited pronounced clustering/multimerization, precluding assignment of a unique precursor CCS distribution for robust filtering and quantitation (Fig. S7). Similar clustering behavior has been reported for other short-chain carboxylates (*e.g.*, HFPO-DA/GenX and PFBA),^38,43^ where a single mobility distribution is not consistently observed; for these analytes, quantitation relied on retention time and accurate mass filters rather than CCS constraints.

Following establishment of analyte-specific LOD, LOQ, and ULOQ, serum concentrations and detection frequencies were evaluated (Fig. 2). Across the panel, LODs ranged from 0.05–5 ng mL^−1^, with generally lower LODs for legacy long-chain analytes and FTS species (ordered from the top), and higher LODs for short-chain carboxylates and select ether-linked replacements (*e.g.*, HFPO-DA/GenX, NaDONA, shown alongside ultra-short chain species towards the bottom of the *y*-axis for compound orientation). This trend is consistent with class-dependent ionization and gas-phase behavior, including fragmentation and clustering that can reduce quantitative sensitivity for certain PFCAs and replacement chemistries. In this study, detection frequencies were highest (>80%) for common legacy PFAS (*e.g.*, PFOS, PFOA, PFNA) and for *N*-MeFOSAA, *N*-EtFOSAA, and 8 : 2 FTS. At the same time, many replacement ether chemistries were detected infrequently (∼10%), likely reflecting a combination of lower exposure prevalence and higher analytical detection limits. Among ultra-short-chain targets, only short-chain sulfonates (TFMS, PFEtS) were observed in serum, despite low LODs observed for these analytes in the FBS matrix.

### Serum PFAS concentrations over time

Because the study sample set (*n* = 36) was designed primarily to evaluate method performance prior to deployment to larger sample sets, the present analysis was not powered for rigorous inference or stratified analyses using available metadata (*e.g.*, sex, age, race, occupational descriptors). Nonetheless, we have observed several molecule-specific associations across the 1992–2006 collection period (Fig. 2, right). Declining time-dependent trends were observed for PFOS (*p* = 0.0004), *N*-EtFOSAA (*p* = 0.0181) and the sum of 10 PFAS (see Fig. 2; *p* = 0.0053). Examination of the relative PFAS composition further suggests that, although PFOS, PFOA, and PFNA remain dominant contributors over time (with decreasing total burden), the proportional contributions of *N*-MeFOSAA, *N*-EtFOSAA, and 8 : 2 FTS diminish in samples collected after ∼2005. For 8 : 2 FTS, many measurements approached the LOQ (0.05 ng mL^−1^). In contrast, despite reduced abundance in later years *N*-MeFOSAA and *N*-EtFOSAA remained frequently detected (LOQ 0.1 ng mL; detection frequency >90%), such that their stacked-bar contributions may appear minimal in the most recent samples even when present at low concentrations. In addition, we found that female subjects had significantly lower levels of PFOS, PFOA, 8 : 2 FTS and the sum of 10 PFAS, consistent with prior reports in the general population (Fig. S8 and SI Excel Sheet).^44–46^ We note that these preliminary findings are again based on a relatively small sample cohort, and further these findings will be re-evaluated in the full cohort study comprised of >600 samples for full statistical characterization.

### Targeted PFAS correlations

To assess correlations between targeted PFAS analytes in this study, serum concentrations were log-scaled and both Pearson (*r*) and Spearman (rho) correlations were performed to assess the potential trends/correlations, and the results are highlighted in Fig. S8. Significant positive correlations were observed for PFOS–PFOA (*r* = 0.81), PFOS–PFHxS (*r* = 0.76), PFHxS–PFOA (*r* = 0.67), and PFNA–PFUdA (*r* = 0.66), as well as between *N*-MeFOSAA and *N*-EtFOSAA (*r* = 0.69).

### Suspect screening results

In addition to targeted quantitation, full-scan data were interrogated for additional PFAS candidates and other chemical classes not included in the quantitative panel. For additional PFAS suspect analysis, we used data from previous IMS studies that reported verified CCS values.^37,39,47^ A curated list of ∼50 PFAS suspects was compiled from these prior reports and screened retrospectively. Four suspect PFAS were detected based on accurate mass and mobility/CCS filtering where applicable: PFECHS, 10 : 2 FTS, FOSAA, and Nafion Byproduct 2. Peak areas for these suspected PFAS were extracted in Skyline. As expected, these PFAS suspects accounted for 3–15% of the overall PFAS signal in each tested sample when compared using peak area (Fig. S9); however, these comparisons should be interpreted cautiously as ionization efficiencies and matrix effects differ across analytes, and peak area does not directly translate to concentration without compound-specific calibration.

**Fig. 3:**
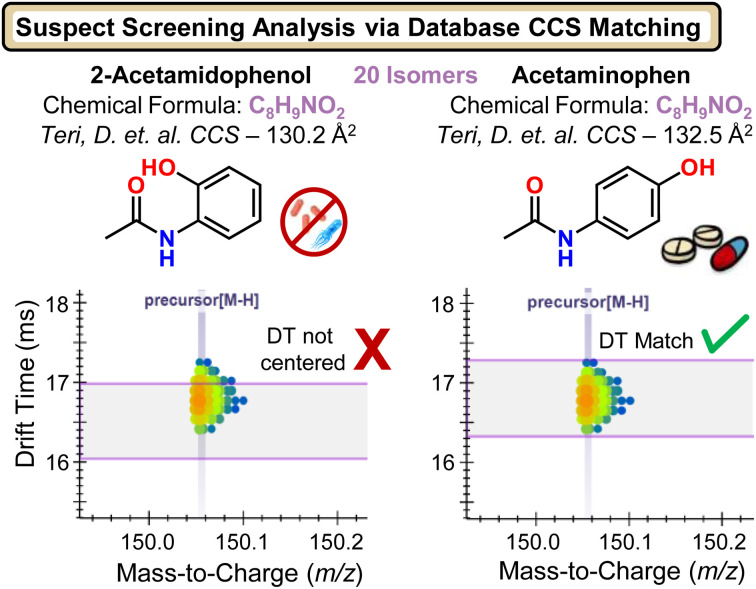
CCS-enabled suspect screening and molecular annotation in Skyline. Example workflow showing how candidate features are prioritized using accurate mass (*m*/*z*) and collision cross section (CCS) agreement, with CCS-based mobility filtering applied to improve selectivity and reduce isomeric ambiguity.

To broaden biomonitoring beyond PFAS, CCS-based mobility filtering was applied using the broad exposomic library published by Teri *et al.*^40^ which provides CCS values for thousands of compounds of interest from both endogenous and exogenous origins. Screening of the ESI- subset enabled tentative annotation of 49 non-PFAS suspect compounds, these molecules met confidence criteria based on accurate mass agreement and CCS/arrival-time filtering. The utility of CCS constraints used in this approach is illustrated in Fig. 3 using an ion observed at *m*/*z* 150.0559, which matched the molecular formula C_8_H_9_NO_2_ with ∼1.3 ppm mass error. While this formula corresponds to many potential isomers (*e.g.*, as indexed in HMDB, potentially 20 divergent structures), the Teri *et al.* library contained a smaller set of 4 candidate structures with CCS values spanning 130.2–133.0 Å^2^. Mobility filtering improved annotation confidence by highlighting candidates whose CCS-predicted arrival time aligned with the observed drift distribution, increasing support for the most plausible isomeric assignment, exemplified in favor of acetaminophen over 2-acetamidophenol.

**Fig. 4:**
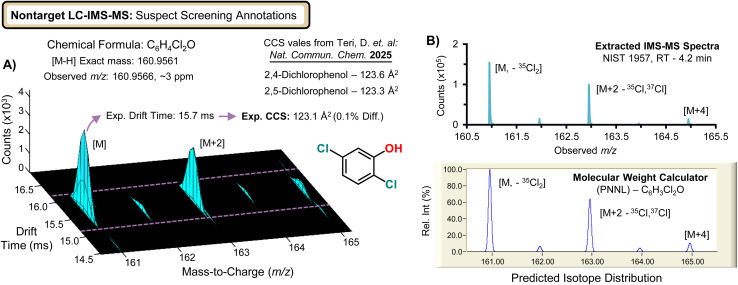
Improved suspect annotation of halogenated species using IMS-MS isotopic constraints. (A) Example feature showing drift-time–aligned isotopic distributions that support molecular annotation of a dichlorophenol with branching ambiguity. The observed isotopic envelope closely matches the theoretical pattern predicted by the PNNL Molecular Weight Calculator (B), increasing confidence in the proposed formula alongside accurate mass and CCS agreement.

Additional confidence in the interpretation of the suspect screening data can be gained by integrating isotopic envelope agreement for halogenated chemicals. Fig. 4 shows a precursor ion signal at *m*/*z* 160.9561 matching formula C_6_H_4_Cl_2_O (∼3 ppm mass error). The observed CCS was in close agreement with 2,5-dichlorophenol (ΔCCS ∼0.1%), while an alternative dichlorophenol positional isomer with 2,4 branching exhibited a CCS mismatch, analogous to the discrimination shown in Fig. 3. The measured isotope pattern further supported the formula assignment *via* the characteristic M+2 signature expected for two chlorine atoms. Collectively, these examples demonstrate how high-resolution IMS-MS enables improved suspect-screening specificity through combined accurate mass, CCS-based mobility filtering, and isotopic pattern constraints, a trait which is particularly valuable when commercial standards are unavailable or difficult to obtain. Across the 49 tentatively identified suspects, compound classes included chloro-/bromophenols, phthalates, fatty acids, and metabolites; complete annotations (retention time, accurate mass and error, candidate formula, CCS) are provided in the SI spreadsheets.

### Integrated correlation analysis across targeted PFAS and SSA detections

**Fig. 5:**
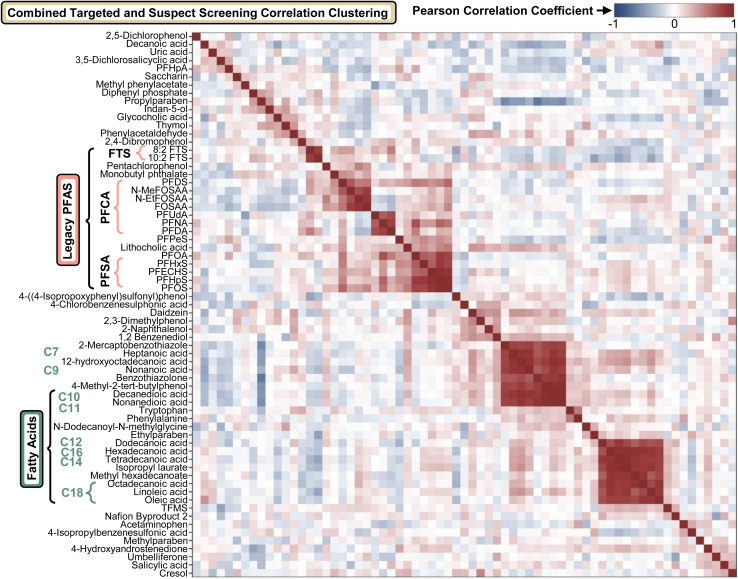
Pearson correlation clustering of targeted PFAS and suspect-screened analytes. Correlations were computed from normalized peak areas and visualized with hierarchical clustering using the average-linkage method. Distinct clusters were observed for legacy PFAS, as well as for fatty acids exhibiting trends consistent with increasing carbon chain length.

To evaluate relationships between targeted PFAS and SSA detections, Pearson correlation clustering was performed using peak areas for both datasets combined (Fig. 5; pairwise correlation matrix in Fig. S10). Legacy PFAS clustered strongly, with additional organization by headgroup (PFCAs *vs.* PFSAs *vs.* FTS). Several emerging PFAS (*e.g.*, TFMS, Nafion Byproduct 2) appeared to cluster distinctly from legacy PFAS, potentially reflecting different source profiles within this small sample set; these patterns will be reassessed in the full PROMETHEUS dataset where increased power and metadata integration (*e.g.*, geography, base assignment, and potential regional manufacturing proximity)^47^ may enable more definitive source attribution. Beyond PFAS, additional clustering trends were observed for medium- to long-chain fatty acids consistent with chain-length-related behavior.

## Conclusions

While targeted LC-MS remains the gold standard for quantitative exposure assessment, the expanding chemical space of PFAS and other compound classes increasingly motivates complementary suspect screening/non-targeted analysis workflows. Here, we demonstrate a scalable serum extraction/analysis workflow that integrates targeted PFAS quantitation and analysis of additional PFAS and other suspect molecules using LC-IMS-MS, a technique that allows for improved selectivity and confidence in annotation of the detected molecular features. The proposed serum extraction protocol is compatible with limited sample volumes (50 µL) and can be completed within ∼1 day depending on batch size. Quantitative performance benchmarked against NIST SRM 1957 supports its suitability for deployment to large sample sets.

A key advantage of the proposed IMS-enabled suspect screening is the addition of the CCS value as an additional molecular annotator that complements accurate mass and isotopic pattern constraints and can reduce ambiguity among isomeric candidates. Importantly, CCS values are translatable across laboratories, enabling shared CCS libraries for prioritization of suspect features when commercial standards are unavailable or difficult to obtain. Although the present pilot study emphasizes workflow development and preliminary profiling, future work will further enhance confidence in suspect annotations through MS/MS fragmentation matching and, where feasible, retention time confirmation using authentic standards. Suspect features identified here will be prioritized for targeted confirmation in the larger set of PROMETHEUS samples and other biomonitoring studies. Collectively, this combined targeted IMS-enabled suspect screening strategy establishes a practical platform for large-scale analysis of archived military serum to investigate exposure patterns and potential exposure-disease relationships in the context of occupationally relevant chemical burdens.

## Ethical statement

The Uniformed Services University of the Health Sciences and the Walter Reed National Military Medical Center Institutional Review Boards reviewed and approved all study protocols under IRB DBS.2024.761, and all study participants provided informed consent.

## Author contributions

The manuscript was written through contributions of all authors and all authors have given approval to the final version of the manuscript. James N. Dodds: conceptualization, software, methodology, formal analysis, validation, data curation, investigation, writing - original draft, visualization. Nikki Barlow: conceptualization, methodology, data curation. Writing - review & editing. Kara M. Joseph: conceptualization, validation, formal analysis, writing - review & editing. Sarah J. Rehm: conceptualization, validation, formal analysis, writing - review & editing. Weihsueh A. Chiu: writing - review & editing, supervision, project administration, funding acquisition. Gang Han: formal analysis, writing - review & editing. Yu-Syuan Luo: formal analysis, writing - review & editing. Kangmin Zhu: conceptualization, methodology, resources, writing - review & editing, supervision, project administration, funding acquisition. Ivan Rusyn: conceptualization, methodology, resources, writing - review & editing, supervision, project administration, funding acquisition. Erin S. Baker: conceptualization, methodology, resources, writing - review & editing, supervision, project administration, funding acquisition.

## Conflicts of interest

The authors declare no competing financial interest.

## Supplementary Material

VA-005-D6VA00088F-s001

VA-005-D6VA00088F-s002

## Data Availability

Raw LC-IMS-MS data files and Skyline documents have been deposited in Panorama Web, a publicly accessible mass spectrometry data repository; the permanent hyperlink is provided in the main text. The supplementary information (SI) Excel files include extracted peak area abundances for targeted and suspect-screened features, along with matrix-matched calibration curve information (generated in Skyline) to enable translation of peak areas into quantified PFAS concentrations and support reuse and reanalysis of the dataset. Supplementary information: LC-IMS-MS settings, Skyline targeted PFAS and suspect screening library, as well as representative calibration curves and correlation matrices. See DOI: https://doi.org/10.1039/d6va00088f.
