# Supplementary material for: Insights into complementary exposomic targeted analysis and suspect screening approaches: a case study examining human serum for chemicals with LC-IMS-MS
Source: Env Sci Adv. 2026 Apr 17;5(5):1306–15. doi: 10.1039/d6va00088f (PMC13088947; doi:10.1039/d6va00088f)
Supplement: VA-005-D6VA00088F-s002 [file VA-005-D6VA00088F-s002.pdf]

Supplementary Information (SI) for Environmental Science: Advances.
This journal is © The Royal Society of Chemistry 2026

Supplementary Materials

"Insights into Complementary Exposomic Targeted Analysis and Suspect Screening Approaches: A Case Study Examining Human Serum for Chemicals with LC-IMS-MS"

James N. Dodds,[1*] Nikki Barlow,[2] Kara M. Joseph,[1] Sarah J. Rehm,[1] Weihsueh A. Chiu,[2] Gang Han,[3] Yu-Syuan Luo,[4] Kangmin Zhu,[5,6,7] Warren Casey,[8] Ivan Rusyn,[2] and Erin S. Baker[1]*

[1]Department of Chemistry, University of North Carolina at Chapel Hill, Chapel Hill, North Carolina, 27514, United States.

[2]Department of Veterinary Physiology and Pharmacology, Texas A&M University, College Station, TX 77843, United States.

[3]Department of Epidemiology & Biostatistics, Texas A&M University, College Station, TX 77843, United States.

[4]Institute of Food Safety and Health, College of Public Health, National Taiwan University, Taipei 100025, Taiwan

[5]John P. Murtha Cancer Center Research Program, Uniformed Services University of the Health Sciences, Bethesda, MD 20817, United States.

[6]Division of Epidemiology and Biostatistics, Department of Preventive Medicine and Biostatistics, Uniformed Services University of the Health Sciences, Bethesda, MD 20814, United States.

[7]Henry Jackson Foundation for the Advancement of Military Medicine, Bethesda, MD 20817, United States.

[8]Division of Program Coordination, Planning, and Strategic Initiatives, U. S. National Institutes of Health, Bethesda, MD 20892, United States.

* Corresponding Author Email: erinmsb@unc.edu

Comments on LC-IMS-MS Data presented in this Work

In this supporting information we provide specific LC-IMS-MS conditions utilized to obtain data and visualizations of the various data strategies and challenges observed in the pilot study. The attached Supporting Information Excel spreadsheet contains various tabs associated with experimental values and calibrations curves associated for targeted PFAS in addition to a CCS library formatted for Skyline import for non-targeted suspect screening analytes profiled in this work.

Table of Contents

P2 – Figures S1 & S2: Detailed extraction protocol for serum analyzed in the main text, and an exemplified EIC of PFAS.

P3 – Figure S3: Detailed LC-IMS-MS settings used for data acquisition.

P4 – Figure S4: Representative calibration curves for targeted PFAS.

P5 – Figures S5 & S6: Assessment of method accuracy and process efficiency.

P6 – Figure S7: Challenges of quantifying TFA via LC-IMS-MS.

P7 – Figure S8: Spearman and Pearson correlation matrix for targeted legacy PFAS.

P8 – Figure S9: Correlation clustering diagram for quantified PFAS across serum samples.

P9– Figure S10: Pairwise correlation matrix for both targeted and suspect screening analytes.

Figure S1: Expanded serum extraction protocol utilized for sample preparation in this work, expanded for more detail as described in the main text, **Figure 1**. This method was optimized using a framework described through internal collaboration with the U.S. EPA, with adjusted volumes to attain lower limits of detection for our platform. A stepwise procedure is provided below.

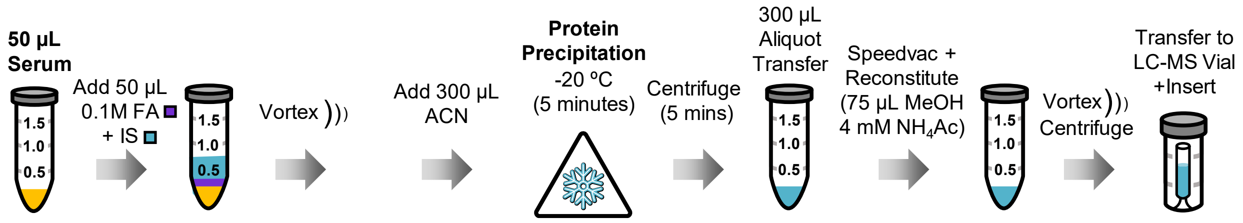

Serum Extraction Procedure

1) Retrieve serum samples from -80ºC freezing and allow to thoroughly melt (typically 30 minutes – 1 hour). This includes all QCs (NIST 1957 and fetal bovine serum (FBS) as appropriate.

2) Transfer 50 µL of serum into a new 2 mL Eppendorf tube, and add 50 µL of 0.1 M formic acid (FA) which has been spiked with internal standard (IS).

3) Vortex the sample and add 300 µL of acetonitrile (ACN). Allow proteins to fully precipitate by freezing the sample at -20 ºC for five minutes.

4) Centrifuge for 5 minutes, then transfer a 300 µL aliquot of supernatant to a new 2 mL Eppendorf tube.

5) Speed vacuum concentrate the sample and reconstitute in 75 µL of methanol buffered with 4 mM ammonium acetate.

6) Vortex and centrifuge the mixture, then aliquot 60 µL into a LC-MS vial with insert for analysis.

Figure S2: Visual illustration of extracted ion chromatograms (EICs) for targeted PFAS used to verify chromatographic performance of the method published by Restek Corporation.[1]

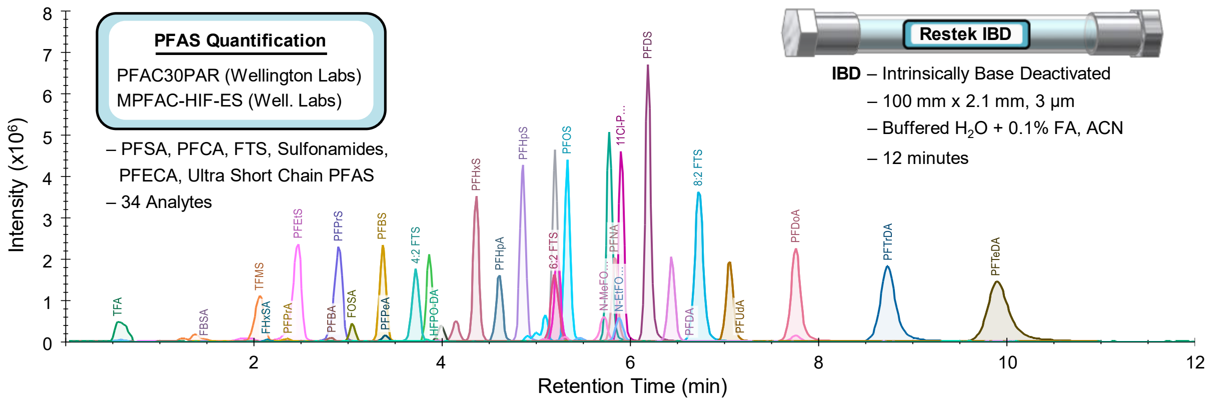

Figure S3: Detailed LC conditions were replicated from a Restek application note from Shun-Hsin Liang titled "Incorporating Ultrashort-Chain Compounds into Comprehensive PFAS Analysis in Waters" and its corresponding publication.[1] IMS-MS conditions for the Agilent 6560 are provided below.

Chromatographic Settings (Agilent 1290 LC)

Parameter	Value
Temperature	40 ºC
Injection Volume	4 µL
Flow Rate	0.4 mL/min
Mobile Phase (A)	Water buffered with 5mM Ammonium Acetate and 0.1% formic acid
Mobile Phase (B)	Acetonitrile
Column	Restek Ultra IBD (3 µm), 100 mm x 2.1 mm (Cat #9175312)
Guard Column	Agilent Eclipse Plus C18, 5 x 2.1 mm (1.8 µm)
**Gradient Table**	
**(minutes)**	**% B**
0	50
7	95
10	95
10.01	50
12.00	50

Agilent 6560 Settings (IMS-MS)

Parameter	Value
**Source Settings**	
Gas Temperature	300 ºC
Drying Gas	5 L/min
Nebulizer	25 psi
Sheath Gas Temp	375 ºC
Sheath Gas Flow	12 L/min
Vcap	4000 V
Nozzle	2000 V
**IMS-MS Settings**	
Mass Range	50-1700 *m/z*
Trap Fill Time	3900 µs
Trap Release Time	150 µs
Frame Rate	0.9 Frame/s
IM Transient Rate	18 Transients/Frame
Max Drift Time	60 ms
Drift Tube Entrance	-1574 V
Drift Tube Exit	-224 V
Rear Funnel Entrance	-217.5 V
Rear Funnel Exit	-45 V
Multiplexing Mode	4 bit

Figure S4: Representative calibration curves for targeted PFAS as analyzed in Skyline. Panels **A** details an EIC for PFOA and corresponding heavy isotopic label standard, and the resulting 1/x weighted calibration curve after normalization to IS (**B**). Panels **C** and **D** illustrate similar results for PFBS. Limits of detection (LOD) were defined as the lowest concentration demonstrating a monotonic increase in signal with concentration and exceeding 3σ of the method blank. Limits of quantification (LOQ) were defined as 10σ above the blank, <20% RSD across replicate extractions, and ≥ LOD. Detailed results for each calibration curve and corresponding LOD, LOQ, and ULOQ are provided in the Supporting Information Excel Sheets.

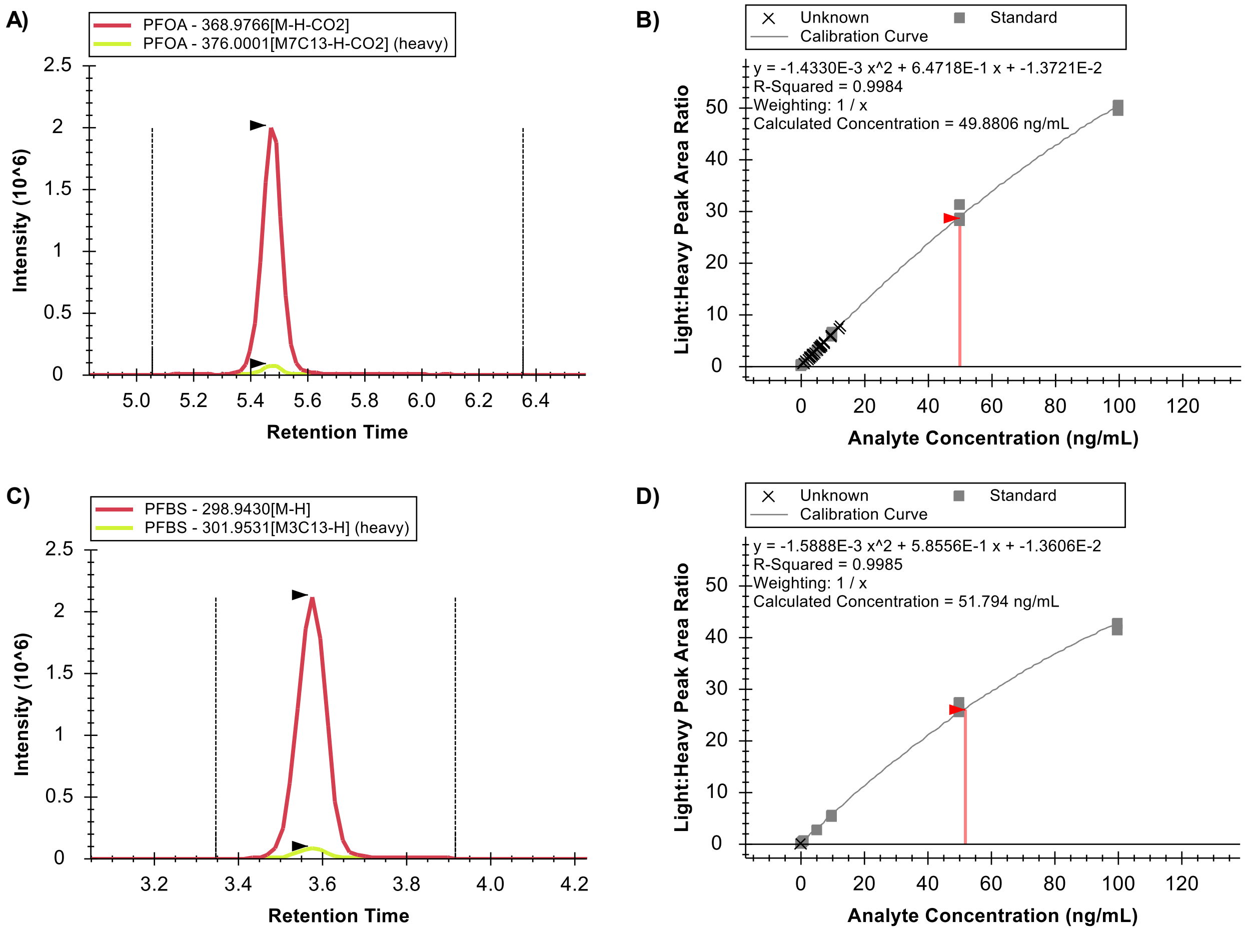

Figure S5: Assessment of method accuracy in correlation to published PFAS values in the NIST 1957 SRM (Human Plasma).

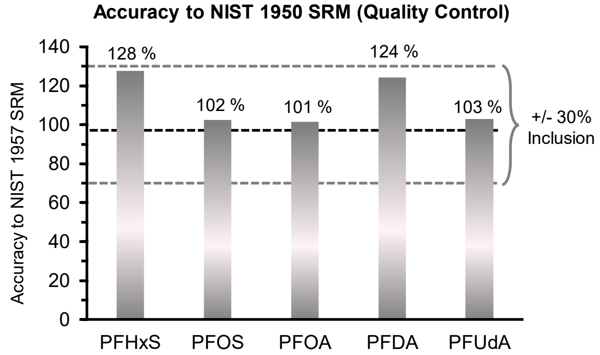

	PFHxS	PFOS	PFOA	PFDA	PFUdA
**NIST Concentrations (ng/mL)**	3.25	10.64	3.27	0.32	0.19
**Observed**	4.15	10.9	3.32	0.40	0.19

Figure S6: Evaluation of method process efficiency for targeted PFAS values extracted in FBS matrix-matched calibration curves. Process efficiency was calculated analogous to Boatman et. al.[2] as follows:

$$\text{Process Efficiency (\%)} = \frac{\text{Peak Area (pre-extraction spike)}}{\text{Peak Area (spiked neat solvent)}} \text{ x } 100$$

While most process efficiencies were between 70-130%, high process efficiencies (200-300%) were in particular for noted particularly for the FOSAA subclass, potentially indicating a headgroup specific-behavior related to the neat solvents or extraction process. Although process efficiency was also high for PFUdA, the excellent accuracy to the NIST 1957 SRM is encouraging for subsequent data analysis.

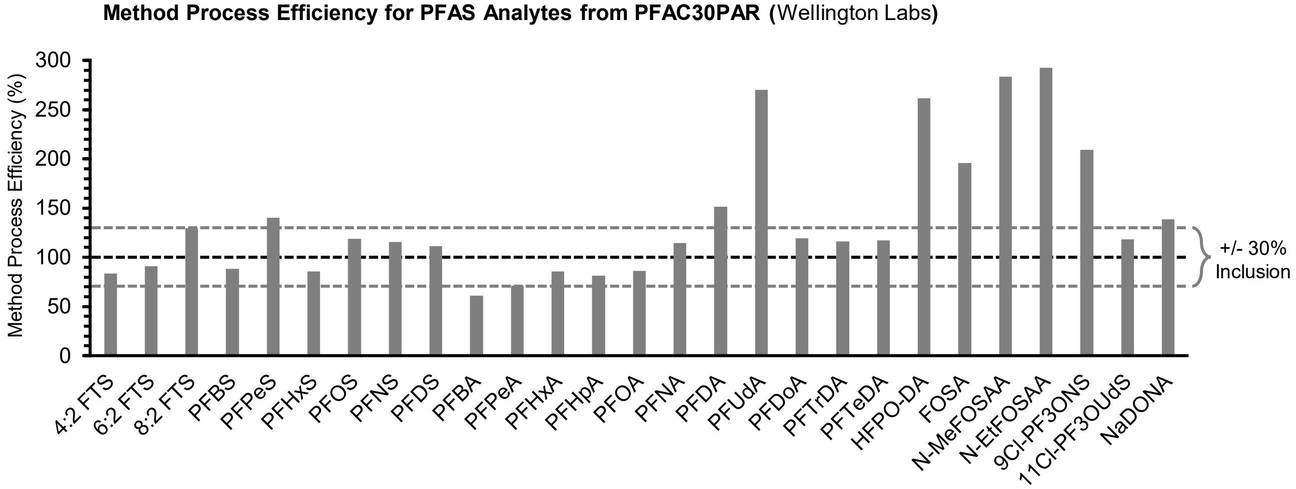

Figure S7: Illustration of the annotation and quantification challenges for trifluoracetic acid (TFA) observed during method development. TFA elutes at the void volume for this chromatographic method, likely a consequence of the differing matrix utilized herein (serum) instead of water analyzed in the manufacturer's application note. In addition to lack of retention, TFA exhibits severe clustering through ionization with ESI noting the adducts described and mobility-alignment of these distributions, precluding CCS filtering efforts for improved data extraction.

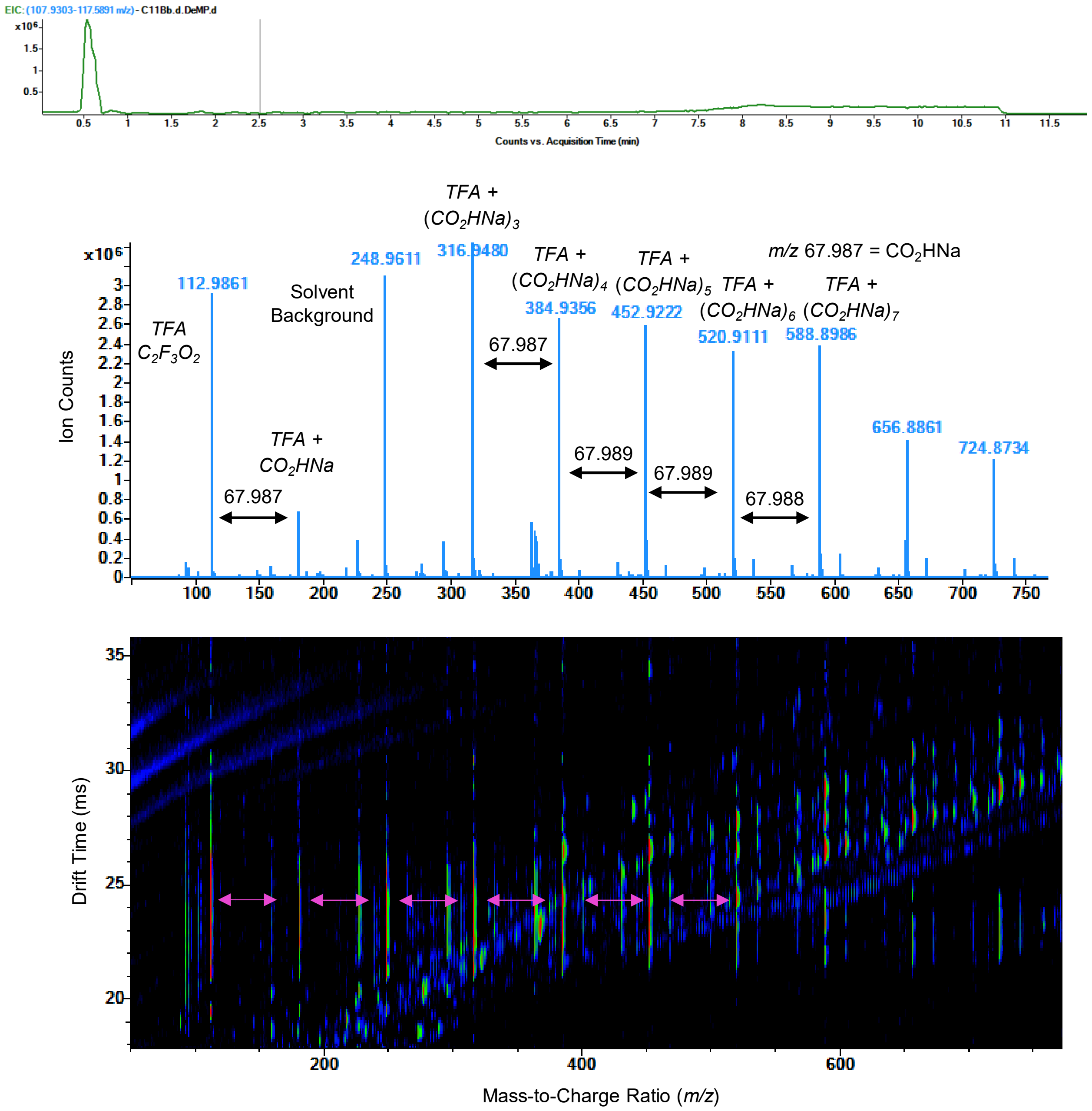

Figure S8: Scatterplot matrix of log10-transformed concentrations with Pearson (r) and Spearman (ρ) correlation coefficients (rho) and associated p-values (p, N=36); red outlines denote statistically significant associations.

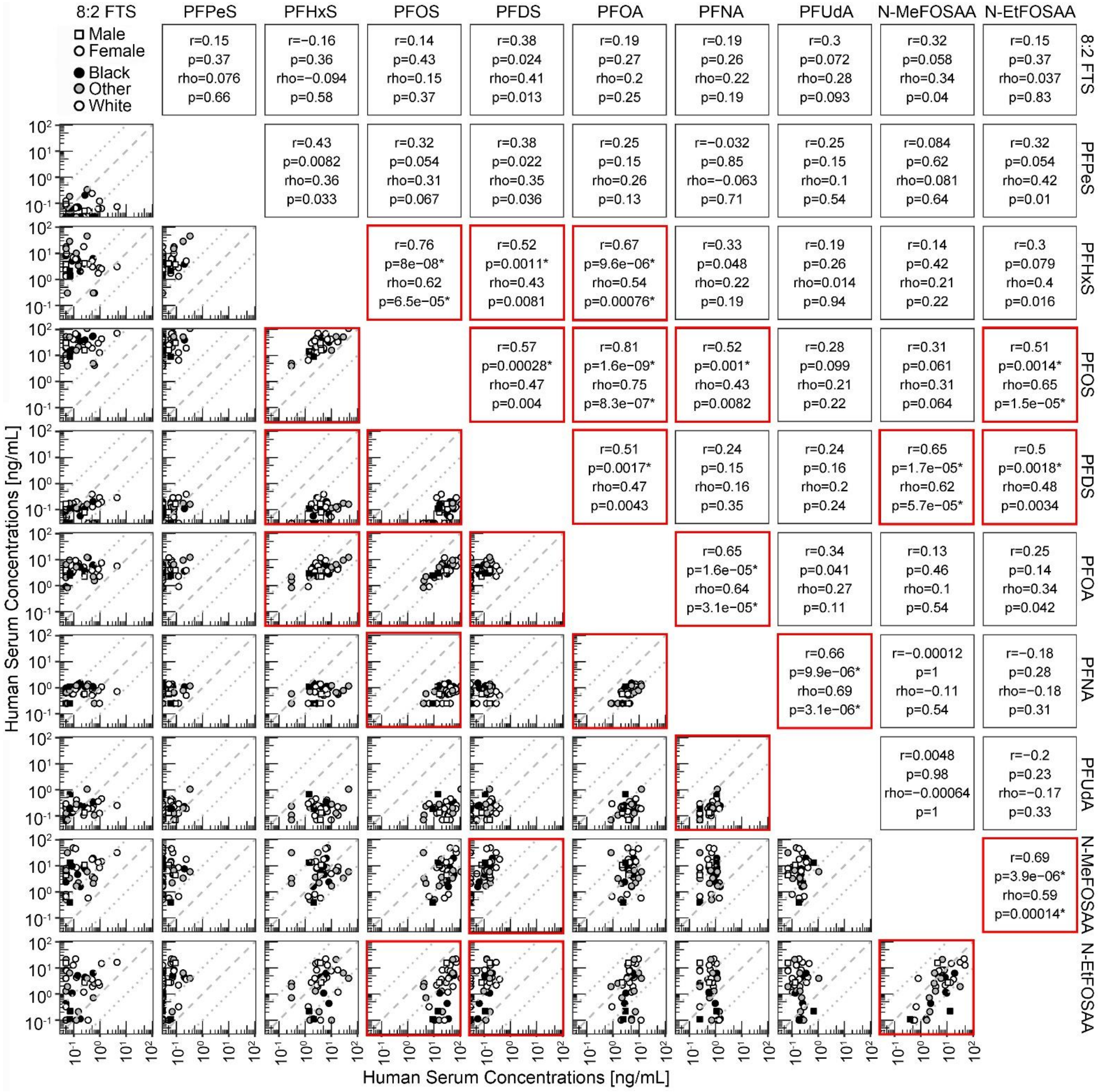

Figure S9: Expanded version of the rightmost panel of Figure 2 from the main text with included stacked bars for both targeted and non-targeted suspect screening PFAS analytes detected in serum. For this plot only peak areas instead of absolute concentrations are reported to promote equivalency between analytes. While this visualization approach does not account for potential differences in ionization efficiency across PFAS analytes, the fraction observed for these analytes is relatively small in proportion to the primary 10 PFAS quantified across the dataset.

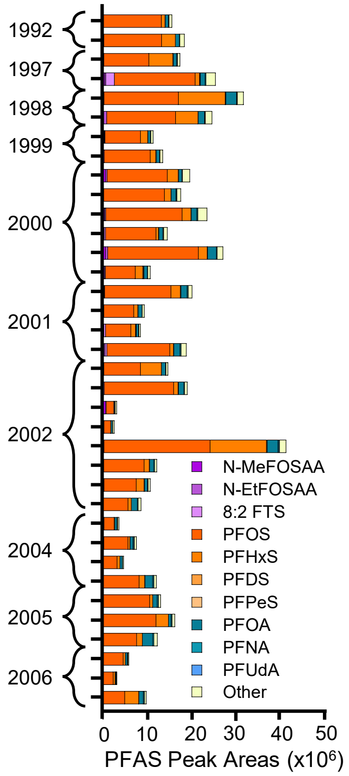

“Other” (PFHpA, FBSA, FHxSA, PFHpS, TFMS, PFECHS, 10:2 FTS, FOSAA)

Figure S10: Pairwise correlation matrix of all analytes assessed in the pilot study, including both targeted PFAS and suspect screening analytes (PFAS and those observed from Teri et. al). To promote equivalency between analytes, raw peak areas were utilized for all chemicals as opposed to exact concentrations for targeted PFAS.

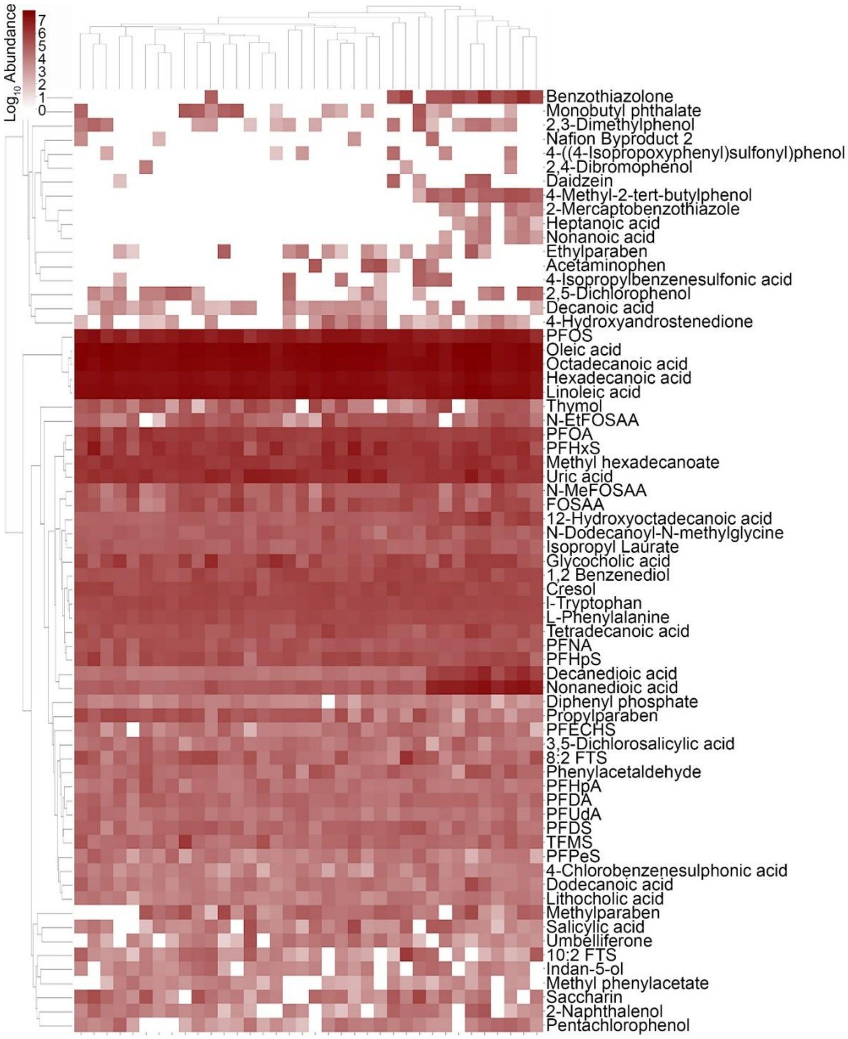

References:

(1) Liang, S.-H. Incorporating Ultrashort-Chain Compounds into Comprehensive PFAS Analysis in Waters. Restek Corporation: Online, 2025; pp 1-15.
(2) Boatman, A. K.; Chappel, J. R.; Polera, M. E.; Dodds, J. N.; Belcher, S. M.; Baker, E. S. Assessing Per- and Polyfluoroalkyl Substances in Fish Fillet Using Non-Targeted Analyses. *Environmental Science & Technology* **2024**, *58* (32), 14486-14495. DOI: 10.1021/acs.est.4c04299.
